# Gene Expression and DNA Methylation Status of Glutathione *S*-Transferase Mu1 and Mu5 in Urothelial Carcinoma

**DOI:** 10.1371/journal.pone.0159102

**Published:** 2016-07-12

**Authors:** Shou-Chieh Wang, Chin-Chin Huang, Cheng-Huang Shen, Lei-Chen Lin, Pei-Wen Zhao, Shih-Ying Chen, Yu-Chiao Deng, Yi-Wen Liu

**Affiliations:** 1 Division of Nephrology, Department of Internal Medicine, Kuang Tien General Hospital, Taichung, 437, Taiwan; 2 Department of Microbiology, Immunology and Biopharmaceuticals, College of Life Sciences, National Chiayi University, Chiayi, Taiwan; 3 Department of Urology, Chiayi Christian Hospital, Chiayi, Taiwan; 4 Department of Forestry and Nature Resources, College of Agriculture, National Chiayi University, Chiayi, Taiwan; University of Navarra, SPAIN

## Abstract

Bladder cancer is highly recurrent after therapy, which has an enormous impact on the health and financial condition of the patient. It is worth developing diagnostic tools for bladder cancer. In our previous study, we found that the bladder carcinogen BBN increased urothelial global DNA CpG methylation and decreased GSTM1 protein expression in mice. Here, the correlation of BBN-decreased GSTM1 and GSTM gene CpG methylation status was analyzed in mice bladders. BBN treatment decreased the protein and mRNA expression of GSTM1, and the CpG methylation ratio of GSTM1 gene promoter was slightly increased in mice bladders. Unlike mouse GSTM1, the human GSTM1 gene tends to be deleted in bladder cancers. Among 7 human bladder cancer cell lines, GSTM1 gene is really null in 6 cell lines except one, T24 cells. The CpG methylation level of GSTM1 was 9.9% and 5-aza-dC did not significantly increase GSTM1 protein and mRNA expression in T24 cells; however, the GSTM5 gene was CpG hypermethylated (65.4%) and 5-aza-dC also did not affect the methylation ratio and mRNA expression. However, in other cell lines without GSTM1, 5-aza-dC increased GSTM5 expression and decreased its CpG DNA methylation ratio from 84.6% to 61.5% in 5637, and from 97.4% to 75% in J82 cells. In summary, two biomarkers of bladder tumor were provided. One is the GSTM1 gene which is down-regulated in mice bladder carcinogenesis and is usually deleted in human urothelial carcinoma, while the other is the GSTM5 gene, which is inactivated by DNA CpG methylation.

## Introduction

Bladder cancer is the seventh most common type of cancer in men worldwide [1] and is fourth among men in the United States [2]. It is not the most lethal cancer, but its high recurrence affects the patient’s quality of life and causes enormous economic burden; therefore, it has a significant impact on healthcare costs. It is known that many risk factors have a high potential to cause bladder cancer, including exposure to smoking [3], arylamines [4], environmental arsenic exposure [5], aging and hereditary influences. Tobacco smoke, the most important risk factor of bladder cancer, contains about 4000 chemicals, many of which are genotoxins including N-nitroso compounds [6] and aromatic amines, e.g. 4-aminobiphenyl (4-ABP) and o-toluidine [7]. When compared with nonsmokers, the odds ratio (OR) of current smokers (7.4) is higher than the OR of former smokers (3.8) [8]. According to the results, quitting smoking immediately is the best suggestion to prevent bladder cancer in smokers.

In 2015, the International Agency for Research on Cancer (IARC) announced that red meat and processed meat (for example, ham, sausages and biltong) are the risk factors of many cancers. Associated with this, it has been reported that carcinogens N-nitrosamines, which are important risk factor for bladder tumor formation [9], exist in bacon and meat products. Besides, N-nitrosamines also play potential roles in chronic urinary tract infection- and schistosomiasis infection-induced bladder cancer [10,11]. Remarkably, N-butyl-N-(4-hydroxybutyl)nitrosamine (BBN), one of the N-nitrosamines, has been identified as an effective and specific bladder carcinogen in some animal studies [12]. Since the carcinogenic potential of BBN is limited to the bladder [13–15], it has been the most commonly-used carcinogen in bladder cancer studies [16–18]. Comparing the gene expression pattern between human and rodents, the BBN-induced B6D2F1 mouse and SD rat bladder cancers are found to be preferentially associated with progression from the non-muscle-invasive to muscle-invasive type of human bladder cancer [19]. Another report has also summarized that the BBN-induced C3H/He mouse bladder cancer gene profile is most similar to invasive human bladder cancer, and nicotinamide can reduce the carcinogenic effect of BBN [20].

By using BBN-induced mouse bladder carcinogenesis and protein 2D electrophoresis analysis, we found that mouse bladder glutathione *S*-transferase Mu1 (GSTM1) protein was down-regulated after BBN treatment [21]. GSTM1 is an important anti-oxidant enzyme in cells, and loss of the GSTM1 gene is known to be correlated with human bladder cancer [22]. Because BBN also increases the whole 5-methylcytosine level in bladder nuclei [18], in this study, we aimed to examine whether GSTM1 gene CpG methylation is responsible for BBN-decreased mouse bladder GSTM1 expression or not. In addition, the correlation of human GSTM expression and DNA methylation was also studied in human bladder cancer cell lines.

## Materials and Methods

### Antibodies

Anti-GSTM1 (GTX113448 for mouse, GTX100298 for human), anti-β-actin (GTX109639) and anti-α-tubulin (GTX112141) antibodies were purchased from GeneTex (Taichung, Taiwan). Anti-NAD(P)H quinone oxidoreductase-1 (NQO1) (ab2346) was purchased from Abcam (Cambridge, UK). Antibodies against p21 (sc-397) and nuclear factor erythroid 2-related factor 2 (Nrf2) (sc-722) were purchased from Santa Cruz (Dallas, Texas, USA). Peroxidase-conjugated secondary antibodies were purchased from Jackson ImmunoResearch (West Grove, PA, USA).

### Animal Treatment for DNA and RNA Extraction

The experiment was approved by the Institutional Animal Care and Use Committee of National Chiayi University. The female C57BL/6 mice were divided into two groups (control and BBN, 10 mice per group) at seven weeks of age. BBN (Tokyo Chemical Industry, Tokyo, Japan) was administered through the drinking water at a final concentration of 300 ppm. The water was renewed twice weekly and lasted for 20 weeks. The mice activity, food, water and room temperature were checked everyday. The mice body weights were measured twice a week, and it shows that no significant difference in control and BBN-treated mice (S1 Fig). Prior to the experimental endpoint, no mice became ill or died. The method of euthanasia was slow CO_2_ inhalation, until no heartbeat, the dissection started. In each group, five mice bladders were collected for DNA extraction, four mice bladders for RNA extraction (2 bladders were combined to 1 sample), half of one mouse bladder for protein extraction and the other half for HE staining.

### Bisulfite Conversion of Genomic DNA and Analysis of DNA Methylation Level in GSTM1 and GSTM5 Gene CpG Island

Five hundred nanograms of genomic DNA was subjected to sodium bisulfite modification using the EZ DNA methylation-Gold^™^ kit (Zymo Research, USA). DNA modified with sodium bisulfite was selectively amplified by PCR using bisulfite specific primers (BSP). The BSP for mouse GSTM1 gene are 5’-GAGTTGAGTTTTTTTAATGATAGATTTTTATAGTTTGG-3’ (forward) and 5’-ATATCAACTAAAAAAAATAACCAAAAAAAACAAAAATC-3’ (reverse), which amplified -293 to 554 bp of the mouse GSTM1 gene (847 bp). The BSP for the human GSTM1 gene are 5’-GGAGAGAAGGYTGAGGGAYA-3’ (forward) and 5’-TRTCCCAARACCCCAARATCAT-3’ (reverse), which amplified -338 to 106 bp of the human GSTM1 gene (444 bp) and also -344 to 99 bp of the human GSTM5 gene (443 bp). PCR products were subcloned into the T&A cloning vector. To determine the CpG methylation status of the 5’ CpG island of each gene, 6–10 clones of each mouse or cell line were randomly picked for sequencing.

### Cell Culture and Treatment

Human bladder cancer cells SV-HUC1, RT4, 5637, TSGH 8301, BFTC 905, T24 and HT 1376 were obtained from the Bioresource Collection and Research Center (Hsinchu, Taiwan), and J82 cells were from ATCC. SV-HUC1 cells were cultured in Ham’s F12 medium, RT4 and T24 cells were maintained in McCoy’s 5A medium, 5637, TSGH 8301 and BFTC 905 cells were cultured in RPMI 1640 medium, and J82 and 1376 cells were maintained in MEM medium. All media were supplied with 10% FBS, 1% penicillin and 1% streptomycin. Cells were incubated in a CO_2_ incubator at 37°C, with 5% CO_2_ and 95% filtered air. 5-Aza-2’-deoxycytidine (5-aza-dC) (Sigma) was dissolved in ethanol and was added to the culture medium in a 1/1000 volume. After 5-aza-dC treatment for 3 days, cells were collected for DNA extraction, RNA isolation and protein lysate preparation.

### Human GSTM1 and GSTM5 Analysis

Genomic DNA was isolated from 7 human bladder cancer lines and SV-HUC1 cells using alkaline lysis methods. The presence or absence of GSTM1 and GSTM5 was detected by multiplex PCR method. The specific primers for GSTM1, forward 5’-GAACTCCCTGAAAAGCTAAAGC-3’ and reverse 5’-GTTGGGCTCAAATATACGGTGG-3’, amplified exons 6 to 7 of the human GSTM1 gene (219 bp) [23]. The specific primers for GSTM5, forward 5’-CACTGCCCCGGTTTTAGTTG-3’ and reverse 5’-CAGGACTGGGAAAGCATCTG-3’, amplified the sequence containing exons 6 and 7 of the human GSTM5 gene (502 bp). Beta-globin was used as a positive control, using the following primers: forward 5’-GAAGAGCCAAGGACAGGTAC-3’ and reverse 5’-CAACTTCATCCACGTTCACC-3’ (268 bp) [23]. DNA (200 ng) was amplified in a total volume of 25 μl, containing 800 nM of each primer, 0.6 unit of *Taq* polymerase and 0.2 mM dNTP. The annealing temperature was 63°C. PCR products were analyzed on 2% agarose gel.

### RT-PCR

Total RNA was isolated from mice bladders and cells. Reverse transcription (RT) was performed on 2 μg of total RNA by 1.5 μM random hexamer and RevertAid^™^ reverse transcriptase (Fermentas), then 1/20 volume of reaction mixture was used for PCR with mouse GSTM1-specific primers (5’GCTCATCATGCTCTGTTACAAC3’, 5’TGTAGCAAGGGCCTACTTGT3’, product size 341 bp), GSTM5-specific primers (5’AGGACTTCATCTCCCGCTTT3’, 5’CTCCCATCTTCTGGCATCAC3’, product size 139 bp), Nrf2-specific primers (5’TCACACGAGATGAGCTTAGGGCAA3’, 5’TACAGTTCTGGGCGGCGACTTTAT3’, product size 182 bp), NQO1-specific primers (5’AAGAGCTTTAGGGTCGTCTTGGCA3’, 5’AGCCTCCTTCATGGCGTAGTTGAA3’, product size 156 bp), p21-specific primers (5’GGAATTGGAGTCAGGCGCAG3’, 5’AGAGTGCAAGACAGCGACAA3’, product size 409 bp) and GAPDH-specific primers (5’CAAGGTCATCCATGACAACTTTG3’, 5’GTCCACCACCCTGTTGCTGTAG3’, product size 496 bp). In a human cell line study, human GSTM1-specific primers (5’ GCACCATGCCCATGATACTG 3’, 5’ TATACGGTGGAGGTCAAGGAC 3’, product size 512 bp), GSTM5-specific primers (5’ AGGACTTCATCTCCCGCTTT 3’, 5’ CTCCCATCTTCTGGCATCAC 3’, product size 139 bp) and GAPDH-specific primers (same as that used in mice) were used. The PCR products were analyzed by 1–2% agarose gel.

### Western Blotting

Analytical 10% sodium dodecyl sulfate (SDS)-polyacrylamide slab gel electrophoresis was performed. Thirty μg protein extracts of each sample were analyzed. For immune-blotting, proteins in the SDS-PAGE gels were transferred to a polyvinylidene difluoride membrane by a trans-blot apparatus. Antibodies against target proteins and β-actin were used as the primary antibodies. Immunoblot analysis was carried out with mouse, rabbit or goat IgG antibodies coupled to horseradish peroxidase. The enhanced chemiluminescence kit and Luminescence Image System (Hansor, Taiwan) were used for detection, and the quantity of each band was determined by the software of MultiGauge.

### Statistical Analysis

The values shown are mean ± SEM. The data were statistically evaluated by performing one-way analysis of variance with SigmaPlot software. P < 0.05 indicated a statistically significant difference.

## Results

### DNA Methylation Level of the GSTM1 Gene CpG Island Was Slightly Increased in BBN-Decreased GSTM1 Expression in Mice Bladders

**Fig 1:**
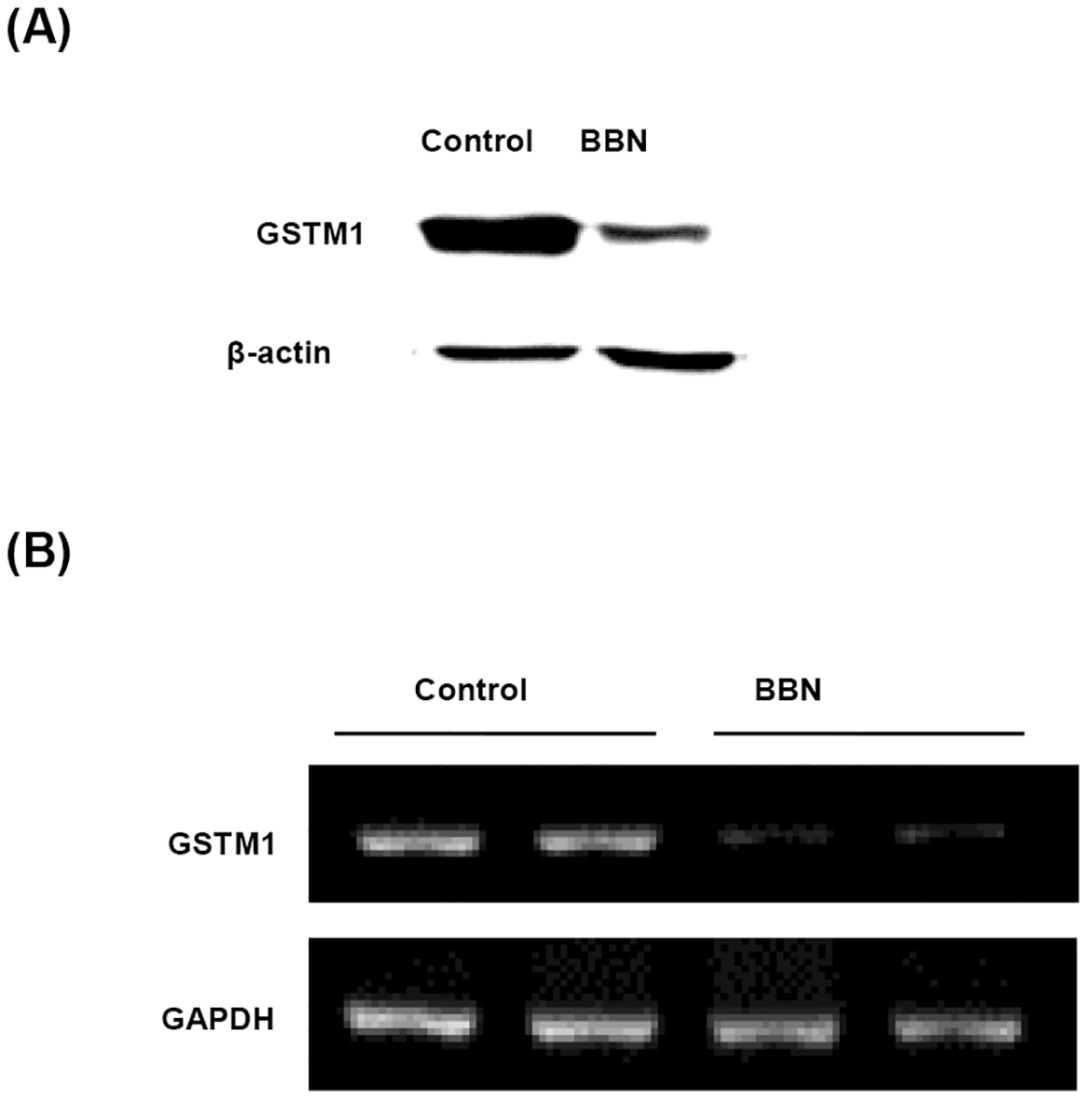
Effect of BBN on the protein and mRNA expression of GSTM1 after treatment for 20 weeks. Mice were treated with or without 300 ppm BBN for 20 weeks. The mice bladders were collected for protein extraction, mRNA and genomic DNA isolation. The expression of GSTM1 protein (A) and mRNA (B) were analyzed by western blot and RT-PCR respectively.

**Fig 2:**
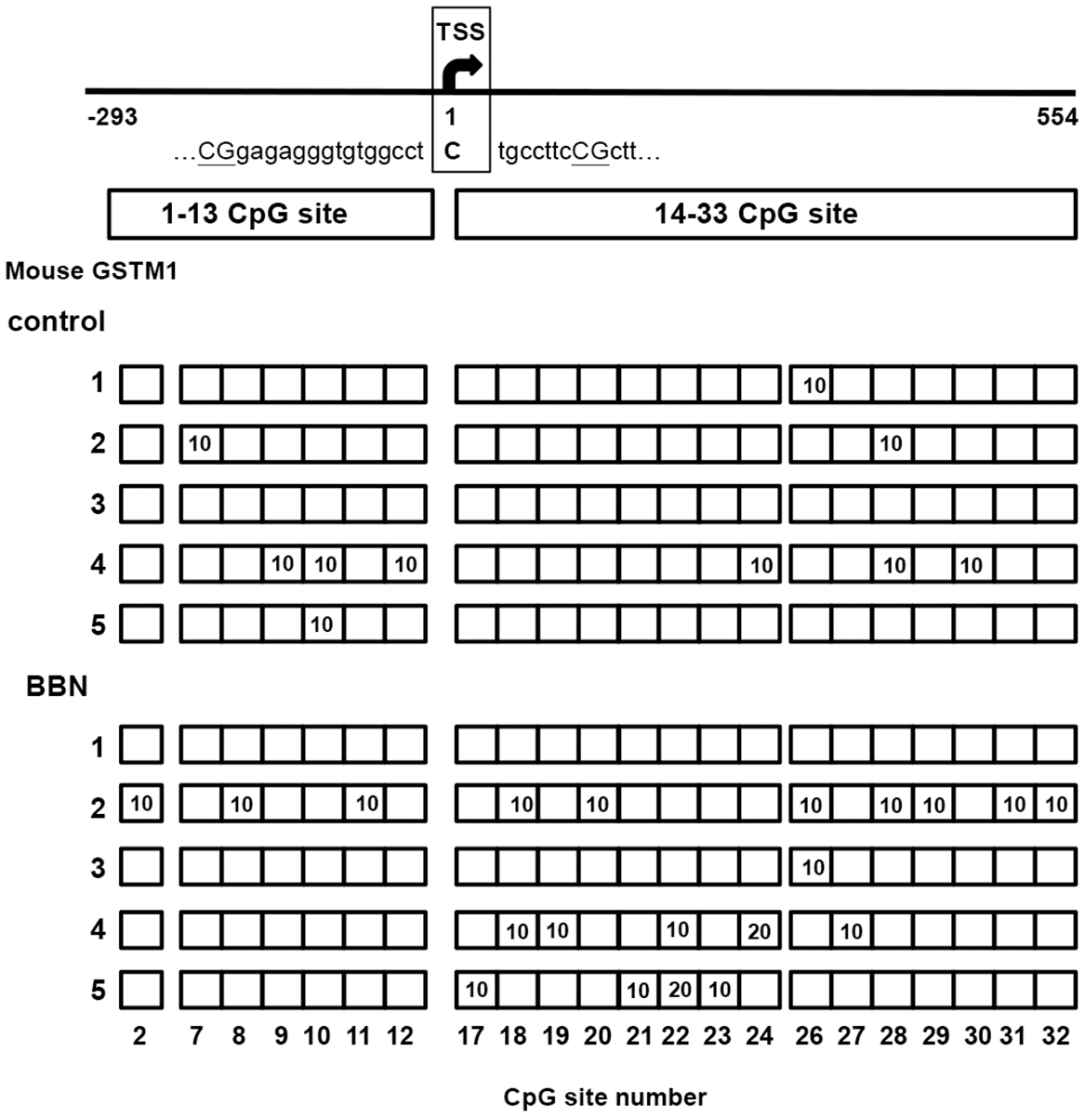
The DNA CpG methylation level of mouse bladder GSTM1 after BBN treatment for 20 weeks. The location of CpG sites and TSS are shown in the upper part. Five control and five BBN-treated mice were analyzed. After PCR and TA cloning, ten clones of each mouse were sequenced. The number in the box indicates the CpG methylation ratio in one CpG site of each mouse.

After BBN treatment for 20 weeks, the mice bladders were collected for protein, mRNA and genomic DNA analysis. The GSTM1 protein and mRNA levels were both down-regulated by BBN treatment (Fig 1), suggesting that BBN decreases GSTM1 expression through transcriptional inhibition in mice bladders. One of the main mechanisms of gene down-regulation is DNA CpG methylation, which leads to gene silencing; therefore, the CpG methylation level was analyzed in the GSTM1 promoter CpG island which included the promoter and 5’-flanking region of the GSTM1 gene. After BBN treatment for 20 weeks, the genomic DNA of mice bladder was extracted and analyzed for the DNA CpG methylation level of the GSTM1 gene. There are 33 CpG sites in the amplified sequence of mouse GSTM1 (-293 to 554 bp), including 13 sites before the transcription start site (TSS) and 20 sites after the TSS. In the 5 control mice, 0 to 6 sites were found to be methylated in each mouse, and the highest methylation frequency of single site was 10%. The average methylation ratio of these 33 sites was 6.1%. In the BBN group, 0 to 10 sites were found to be methylated in each mouse, and the highest methylation frequency was 20% (Fig 2). The average methylation ratio of these 33 sites was 13.3%, which was slightly higher than the control mice. Therefore, DNA CpG methylation might contribute a minor part of BBN-inhibited GSTM1 gene expression in mice bladders.

### The Protein Quantity of Transcription Factor Nrf2 Didn’t Change in BBN-Decreased GSTM1 Mice Bladders

**Fig 3:**
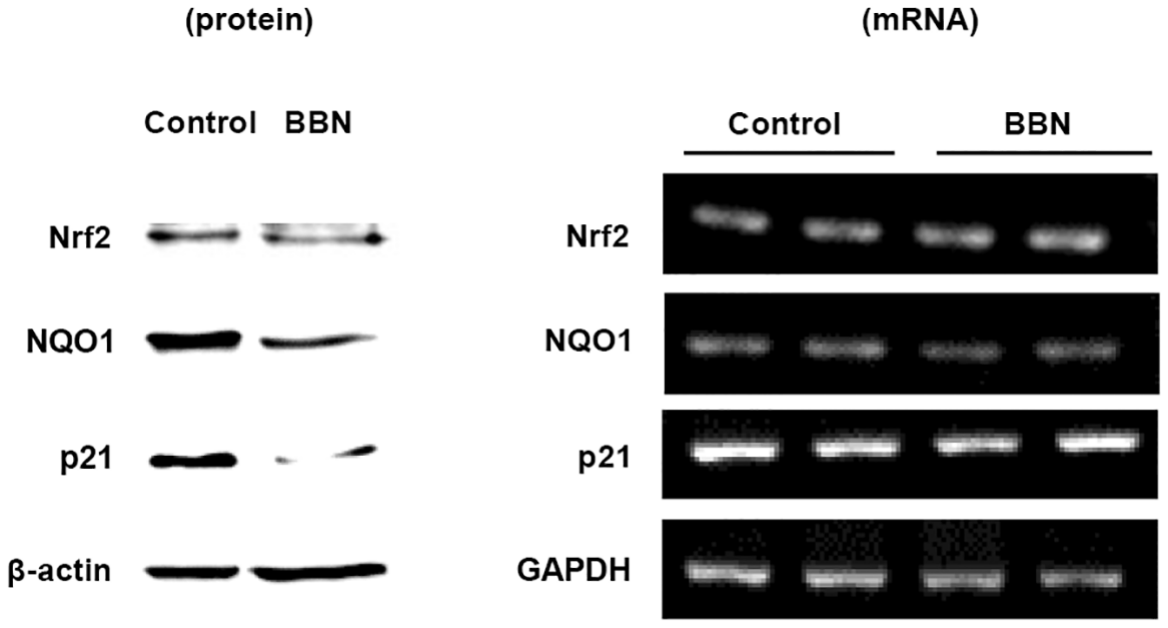
Effect of BBN on the protein and mRNA expression of Nfr2, NQO1 and p21 after treatment for 20 weeks. Mice were treated with or without 300 ppm BBN for 20 weeks. The mice bladders were collected for protein extraction, mRNA and genomic DNA isolation. The expression of protein and mRNA was analyzed by western blot and RT-PCR respectively.

Nrf2, a ubiquitous transfection factor for phase 2 enzymes including GSTM1, was analyzed in the mice samples. It showed no change in either protein or mRNA expression by BBN treatment (Fig 3). Another phase 2 enzyme NQO1 was also analyzed and just like GSTM1, the protein and mRNA expression were also decreased in BBN-treated mouse bladders (Fig 3). We also assayed the change of p21, a cell cycle inhibitor which is unrelated to phase 2 enzymes and decreased p21 expression is associated with bladder cancer stage [24]. Its protein expression was also decreased in the BBN-treated mouse bladder, while its mRNA expression was not affected by BBN (Fig 3). Therefore, BBN-mediated GSTM1 and NQO1 decrease is mediated by transcriptional repression, but it is not true for the p21 protein. In the same conditions, protein and RNA levels of Nrf2 were not affected, which suggests that total quantity of Nrf2 protein does not contribute to the BBN-decreased GSTM1 and NQO1 expression. The protein expression change of NQO1 and p21 were also confirmed by another batch study (S2 Fig). On the other hand, the mRNA expression change of GSTM1 (Fig 1), NQO1, Nrf2 and p21 (Fig 3) were similar to Kim‘s report (NCBI GEO GSE21636 data) [20].

### The Expression Status of Human GSTM1 Gene in Various Human Urothelial Cell Lines and Its DNA Methylation Status in T24 Cells

**Fig 4:**
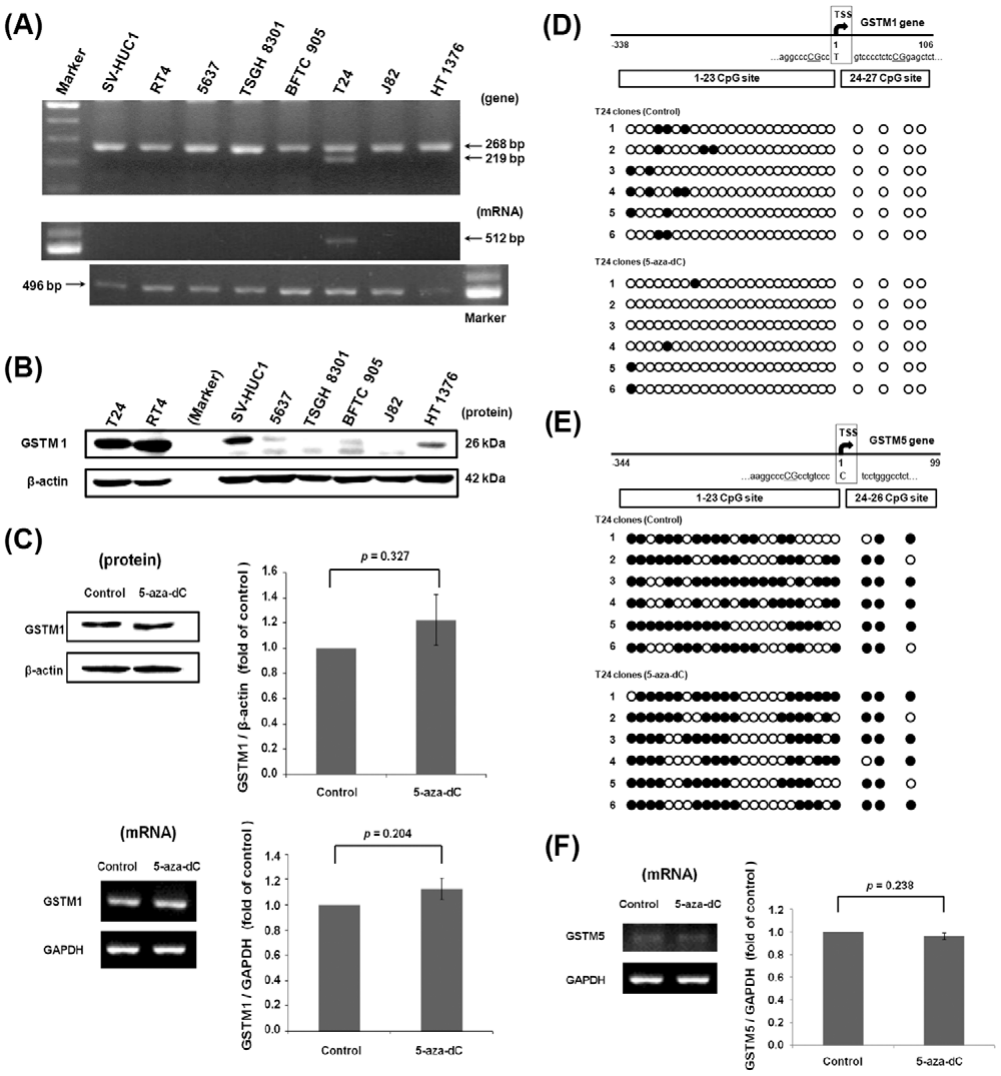
GSTM1 gene expression in human bladder cell lines. (A) The GSTM1 gene and mRNA expression in 8 human bladder cell lines. The PCR product of 268 bp is from the beta-globin gene (positive control), and 219 bp is from the GSTM1 gene. The RT-PCR product of 512 bp is from GSTM1 mRNA, 469 bp is from GAPDH mRNA (positive control). (B) Western blot result using antibody against GSTM1 in 8 human bladder cell lines. Beta-actin was an internal control. (C) Effects of 5-aza-dC on the expression of GSTM1 in T24 cells. The T24 cells were treated with 10 μM 5-aza-dC for 3 days. GSTM1 expression was then analyzed by western blot (with a β-actin loading control) and RT-PCR (with a GAPDH internal control). (D) The DNA CpG methylation level of human GSTM1 in T24 cells. The location of CpG sites and TSS are shown in the upper part. After PCR and TA cloning, six clones were randomly selected for sequencing. Each circle presents one CpG site. The black circle indicates a methylated CpG site, and an open circle denotes an unmethylated CpG site. (E) The DNA CpG methylation level of human GSTM5 in T24 cells. (F) Effects of 5-aza-dC on the expression of GSTM5 in T24 cells.

According to the UniGene protein alignment, the human protein most similar to mouse GSTM1 is human GSTM1, which has 85.3 identities. However, different from mice, the human GSTM1 gene has been reported to be deleted in approximately 50% population, and the GSTM1-null genotype is reported to be more susceptible to bladder cancer [22]. Therefore, the existence of the GSTM1 gene and mRNA was checked here in 8 bladder cell lines including 7 cancer types and one SV40-transformed normal cell type. The data showed that only one cancer cell type, T24, possessed the GSTM1 gene and mRNA, while 6 other cancer cell lines and SV-HUC1 belonged to the GSTM1-null genotype (Fig 4A). Although the GSTM1 gene is only present in T24 cells, the anti-GSTM1 antibody could react with a suspicious band at around 26 kDa in those cell lines without the GSTM1 gene, especially in RT4 cells (Fig 4B). After treatment with BBN, GSTM1 mRNA expression didn’t change in T24 cells (S3 Fig). It indicates that BBN treatment *in vitro* maybe different from *in vivo* because liver metabolism is deficient in cell culture [25]. In order to determine whether the human GSTM1 gene is silenced by DNA methylation or not, T24 cells were chose and then treated with a DNA methyltransferase inhibitor 5-aza-dC to investigate the GSTM1 expression. After 5-aza-dC treatment for 3 days, the protein and mRNA expression of GSTM1 was slightly increased with 1.22- and 1.13-fold, but without a significant difference (Fig 4C). Moreover, the DNA CpG methylation level of the human GSTM1 gene was analyzed and shown in Fig 4D. There are 27 CpG sites in the amplified sequence of GSTM1 (-338 to 106 bp), including 23 sites before TSS and 4 sites after TSS. After reading 6 clones, 2 to 4 sites were found to be methylated in each clone, and the average methylation ratio of these 27 sites was only 9.9%. The ratio was decreased to 2.5% by 5-aza-dC treatment. These results indicate that GSTM1 gene is not heavily silenced by DNA CpG methylation in T24 cells, and the methylation inhibitor slightly changed the DNA methylation status and expression of the GSTM1 gene.

### The Expression Status of Human GSTM5 Gene and Its DNA Methylation Status in T24, 5637 and J82 Cells

**Fig 5:**
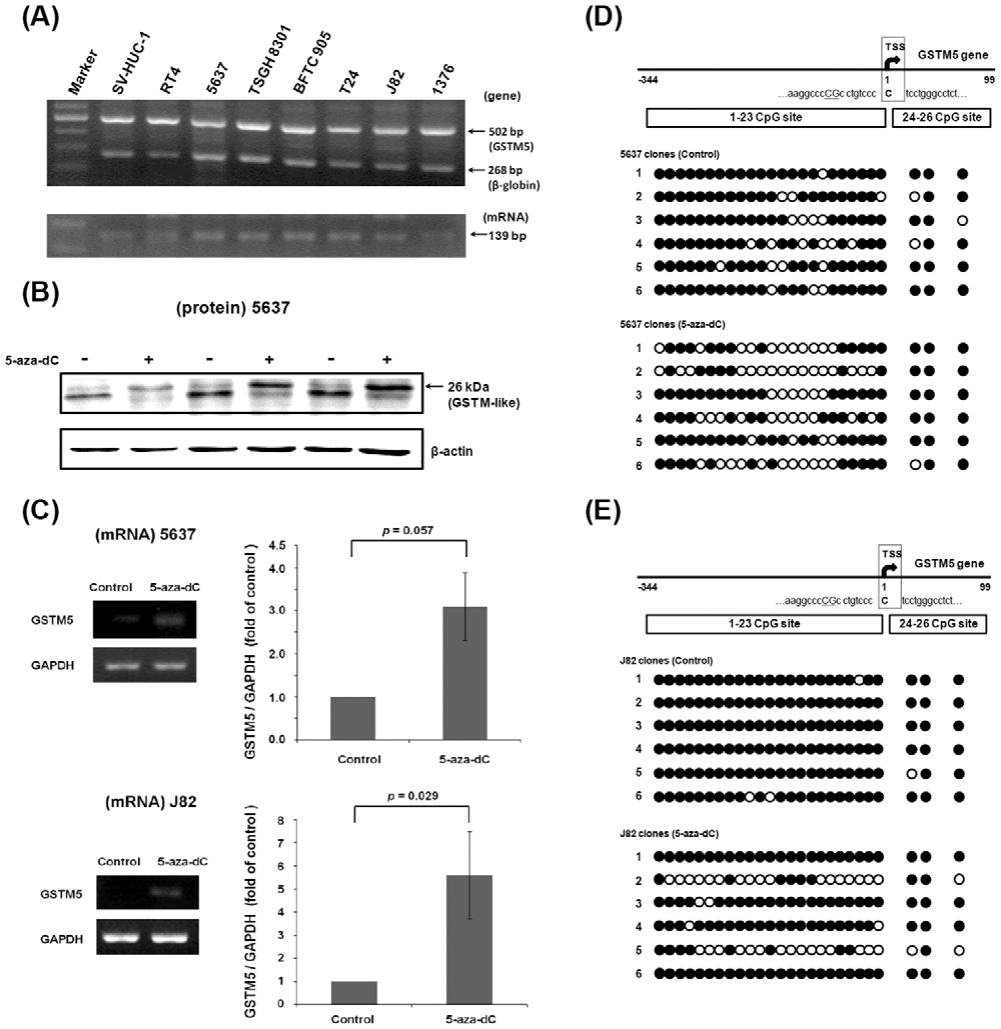
GSTM5 gene expression in human bladder cell lines. (A) The GSTM5 gene expression in 8 human bladder cell lines. The PCR product of 268 bp is from the beta-globin gene (positive control), and 502 bp is from the GSTM5 gene. The RT-PCR product of 139 bp is from GSTM5 mRNA, the positive control GAPDH is the same one in Fig 4A. (B) Western blot using antibody against GSTM1 in 5637 cells. Cells were treated with or without 5-aza-dC for 3 days, with beta-actin as an internal control. (C) Effects of 5-aza-dC on GSTM5 mRNA expression in 5637 and J82 cells. Cells were treated with 10 μM 5-aza-dC for 3 days. GSTM5 expression was analyzed by RT-PCR with a GAPDH internal control. (D and E) The DNA CpG methylation level of human GSTM5 in 5637 cells and J82 cells. Each circle presents one CpG site. The black circle denotes a methylated CpG site, and an open circle indicates an unmethylated CpG site.

When reading the CpG methylation sequence of the GSTM1 gene (-338 to +106 bp) in T24 cells, we found that GSTM1 BSP primers also amplified the GSTM5 CpG island region (-344 to +99 bp) (Fig 4E). In contrast to GSTM1, the CpG methylation level of GSTM5 gene appeared high (65.4%) in untreated T24 cells. After 5-aza-dC treatment for 3 days, the ratio was 63.5%, which is similar to that of the control. GSTM5 RNA expression also was checked by RT-PCR, which shows no significant change (Fig 4F). Because the GSTM5 gene all existed in 7 bladder cancer cell lines and SV-HUC-1 cells (Fig 5A), the expression and regulation of GSTM5 was also analyzed in other cell lines. By western blot analysis, 5-aza-dC treatment induced a 26 kDa band increase using anti-GSTM1 antibody detection in 5637 cells (Fig 5B). As it is known that the GSTM1 gene is deleted in 5637 cells, the 26 kDa band might reflect other GSTM family proteins, like GSTM5. Therefore, the GSTM5 mRNA was analyzed by a pair of GSTM5-specific primers which do not amplify other GSTMs. This showed that GSTM5 mRNA was increased by 5-aza-dC treatment in 5637 (3.09-fold) and J82 cells (5.6-fold) (Fig 5C). Using the same primers as for GSTM1 BSP, the CpG methylation level of GSTM5 gene was analyzed. After 5-aza-dC treatment, the level was decreased from 84.6% to 61.5% in 5637 cells (Fig 5D), and from 97.4% to 75% in J82 cells (Fig 5E). This suggests that GSTM5 gene expression is regulated by DNA CpG methylation in 5637 and J82 cells, but not in T24 cells. The GSTM5 mRNA expression was also analyzed after BBN treatment in T24 and 5637 cells. It shows that like GSTM1, GSTM5 mRNA expression didn’t change after BBN treatment (S3 Fig).

## Discussion

Some reports have summarized that smoking increases the risk of bladder cancer in people with GSTM1-null genotype [22,26]. This suggests that without the detoxifying function of GSTM1, the carcinogen(s) in smoking may not be metabolized easily, which aggressively damages the urothelium. Although there is no GSTM1-null genotype in mice, BBN-decreased GSTM1 protein (Fig 1A) should contribute to the decrease of GSTM1 activity in mice bladders, which may play a critical role in the promotion of BBN-induced bladder carcinogenesis.

After analyzing the 33 CpG methylation sites between -293 to 554 bp of the mouse GSTM1 gene, BBN slightly increases the methylation level (Fig 2), which explains part of the decrease in mRNA and protein levels (Fig 1). However, BBN-decreased GSTM1 gene transcriptional activity might be also caused by other factor(s). Because GST belongs to phase 2 detoxifying enzymes, which are probably activated by Nrf2 [27], the Nrf2 mRNA and protein were also analyzed. This shows that neither mRNA nor protein is affected by BBN (Fig 3), which means that the total quantity of Nrf2 is not responsible for BBN-decreased GSTM1 gene expression in mice. In another study using Keap1 and Nrf2 knockout tools, the results indicate that GSTM3 gene expression is inducible by Nrf2 nuclear translocation, yet GSTM1 is a Nrf2 basal-only target, and not an inducible gene [28]. Another GST family molecule, GSTP, is found to be induced by oltipraz in wild-type but not Nrf2 knockout mice bladders following 2-week BBN treatment, and Nrf2 plays a role in preventing BBN-induced bladder cancer [29]. In spite of neither the total Nrf2 mRNA nor protein level being decreased by BBN in the mouse bladder (Fig 3), whether Nrf2 nuclear translocation or other factor(s) related to BBN decreased GSTM1 gene activity requires further investigation. Human GSTM1 gene expression is also slightly affected by decreasing DNA methylation level (Fig 4C). Because GSTM1 gene CpG methylation is kept at a low rate in T24 cells (Fig 4D), this may explain the minor effect of the demethylation agent 5-aza-dC (Fig 4C and 4D). In fact, the GSTM1 gene activity in humans is mainly inhibited by gene loss [30], and the GSTM1-null status is associated with a modestly increased risk of bladder cancer [22].

In our previous study, we found that GSTM1 mRNA and protein levels were increased by 5-aza-dC treatment in human bladder cancer 5637 cells [21]. However, the GSTM1 gene of 5637 cells was proved to be deleted in this study (Fig 3A). It is known that GSTM family comprises 5 class genes (GSTM1-5) [31], and we found afterwards that the PCR primers used in the previous study were not specific to GSTM1, it might also amplify GSTM2, 4 and 5. Because the protein similarity between GSTM families is high, antibodies against GSTM1 may also recognize other GSTM members in GSTM1-null cell lines (Fig 3B). Although the GSTM1 gene is deleted in 5637 cells, a faint 26 kDa band could be detected and increased by 5-aza-dC treatment (Fig 5B) [21]. This suggests that other GSTM, but not GSTM1, might be regulated by DNA methylation. After the analysis of GSTM5, it suggests that 5-aza-dC does increase GSTM5 mRNA expression (Fig 5C) and decreases the GSTM5 DNA CpG methylation level in 5637 (Fig 5D) and J82 cells (Fig 5E). In addition to GSTM5, human GSTM2 has been found to be silenced by promoter hypermethylation in lung cancer cells [32], and GSTM2, 3 and 5 promoter hypermethylation was also found in Barrett’s adenocarcinoma [33]. In a global DNA methylation analysis of treatment-naïve bladder cancer patients, the DNA methylation in the GSTM2 and GSTM3 promoter was found to be higher in invasive tumors than in non-invasive types [34]. Analysis of the relationship between DNA methylation and GSTM2 and 3 is interesting and ongoing in our laboratory.

Apart from GSTM1, the decreased p21 protein caused by BBN (Fig 3) may also play a role in bladder carcinogenesis. However, different from GSTM1, the mechanism of BBN-decreased p21 is not mediated by transcriptional inhibition (Fig 3). Cyclin-dependent kinase (CDK) inhibitor 1, also known as p21, belongs to a CIP/KIP family which inhibits various CDKs including CDK1, 2, 4 and 6 and then blocks cell cycle progression. In p21 gene knockout mice, tumor progression has been accelerated under the carcinogen stimulation in the colon [35]. It is known that the p21 gene is directly transcriptionally activated by p53 to induce cell cycle arrest in response to DNA damage [36]. However, p21 is degraded by various pathways under low-dose UV irradiation including glycogen synthase kinase 3 beta phosphorylation (ser114)-proteasome [37], PCNA-dependent CRL4^cdt2^ ubiquitination [38] and the LKB1-NUAK1 phosphorylation pathway [39]. In addition to UV irradiation, polycyclic aromatic hydrocarbon carcinogens induce p21 mRNA by p53-induced transcriptional activation while promoting p21 protein degradation by the proteasome [40]. In this study, it demonstrates that carcinogen BBN did not increase p21 mRNA, but instead reduced the p21 protein, which is most likely due to promoted degradation by the proteasome and is worth further investigation.

Based on the evidence revealed in our study, three conclusions are drawn for the first time: the first is that BBN-induced mouse GSTM1 down-regulation is caused by transcription inhibition and DNA CpG methylation plays a small role in the mechanism. Second, the GSTM1 gene is always deleted in human bladder cancer cells. In the cells without a GSTM1 gene, the GSTM5 gene is usually heavily methylated and re-activated by DNA methyltransferase inhibitor. Third, BBN also decreases p21 protein expression, which is not mediated by transcription inhibition. In summary, down-regulation of the GSTM1 gene and the p21 protein might act as biomarkers in nitrosamines-induced bladder carcinogenesis in mice, while GSTM1 null and GSTM5 hypermethylation might act as bladder cancer biomarkers in humans.

## Supporting Information

S1 Fig Mice body weight records. Twenty mice (10 mice/group) body weights were measured and recoded twice a week. The values shown are mean ± SEM.
(PDF)

S2 Fig NQO1 and p21 protein expression of mice bladders with or without 300 ppm BBN treatment for 20 weeks. There are 12 samples (6/group) for NQO1 analysis, and 8 samples (4/group) for p21 analysis. The values shown are mean ± SEM.
(PDF)

S3 Fig GSTM1 and GSTM5 mRNA expression of T24 and 5637 cells with or without BBN treatment. Cells were treated with or without 1.5 mM BBN for 2, 7 and 14 days.
(PDF)
