# Supplementary material for: Gene Expression and DNA Methylation Status of Glutathione S-Transferase Mu1 and Mu5 in Urothelial Carcinoma
Source: PLoS One. 2016 Jul 12;11(7):e0159102. doi: 10.1371/journal.pone.0159102 (PMC4942074; doi:10.1371/journal.pone.0159102)
Supplement: S1 Fig — Twenty mice (10 mice/group) body weights were measured and recoded twice a week. The values shown are mean ± SEM. (PDF) [file pone.0159102.s001.pdf]

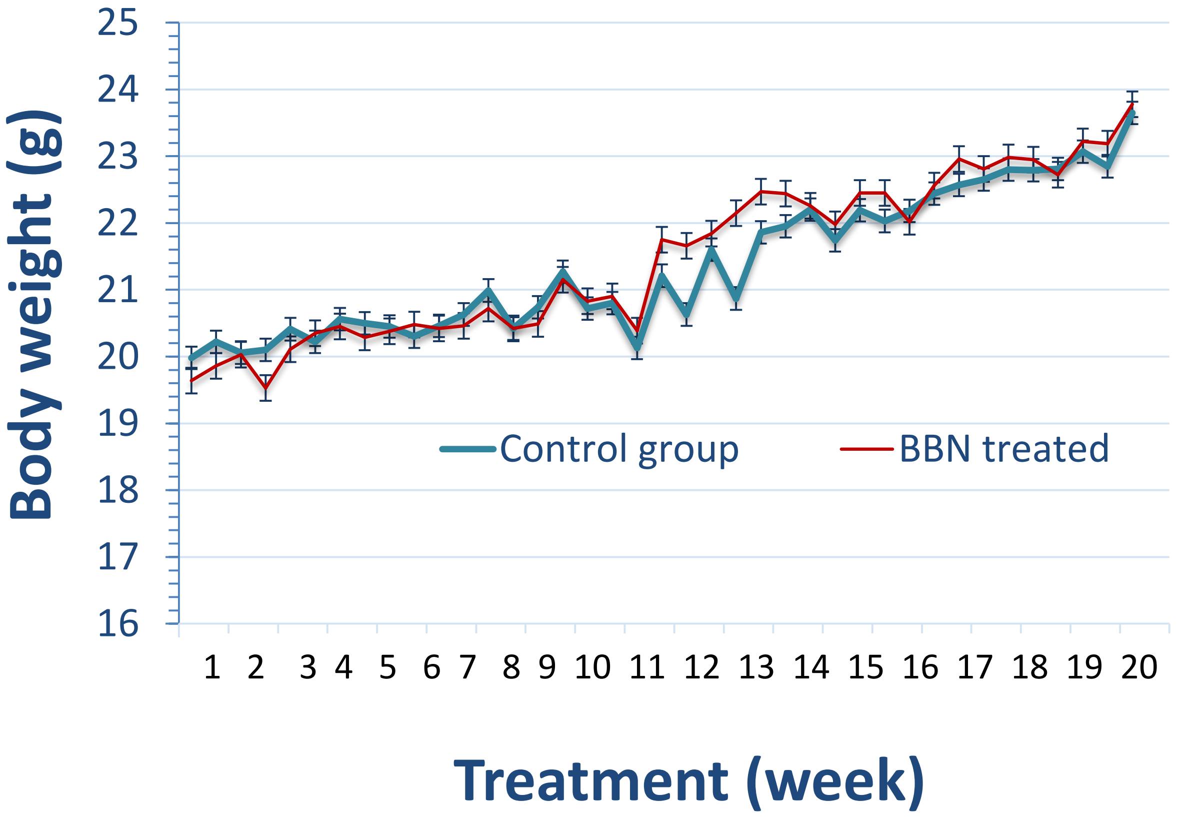

S1 Fig. Mice body weight records. Twenty mice (10 mice/group) body weights were measured and recoded twice a week. The values shown are mean ± SEM.
