# Supplementary material for: Gene Expression and DNA Methylation Status of Glutathione S-Transferase Mu1 and Mu5 in Urothelial Carcinoma
Source: PLoS One. 2016 Jul 12;11(7):e0159102. doi: 10.1371/journal.pone.0159102 (PMC4942074; doi:10.1371/journal.pone.0159102)
Supplement: S2 Fig — There are 12 samples (6/group) for NQO1 analysis, and 8 samples (4/group) for p21 analysis. The values shown are mean ± SEM. (PDF) [file pone.0159102.s002.pdf]

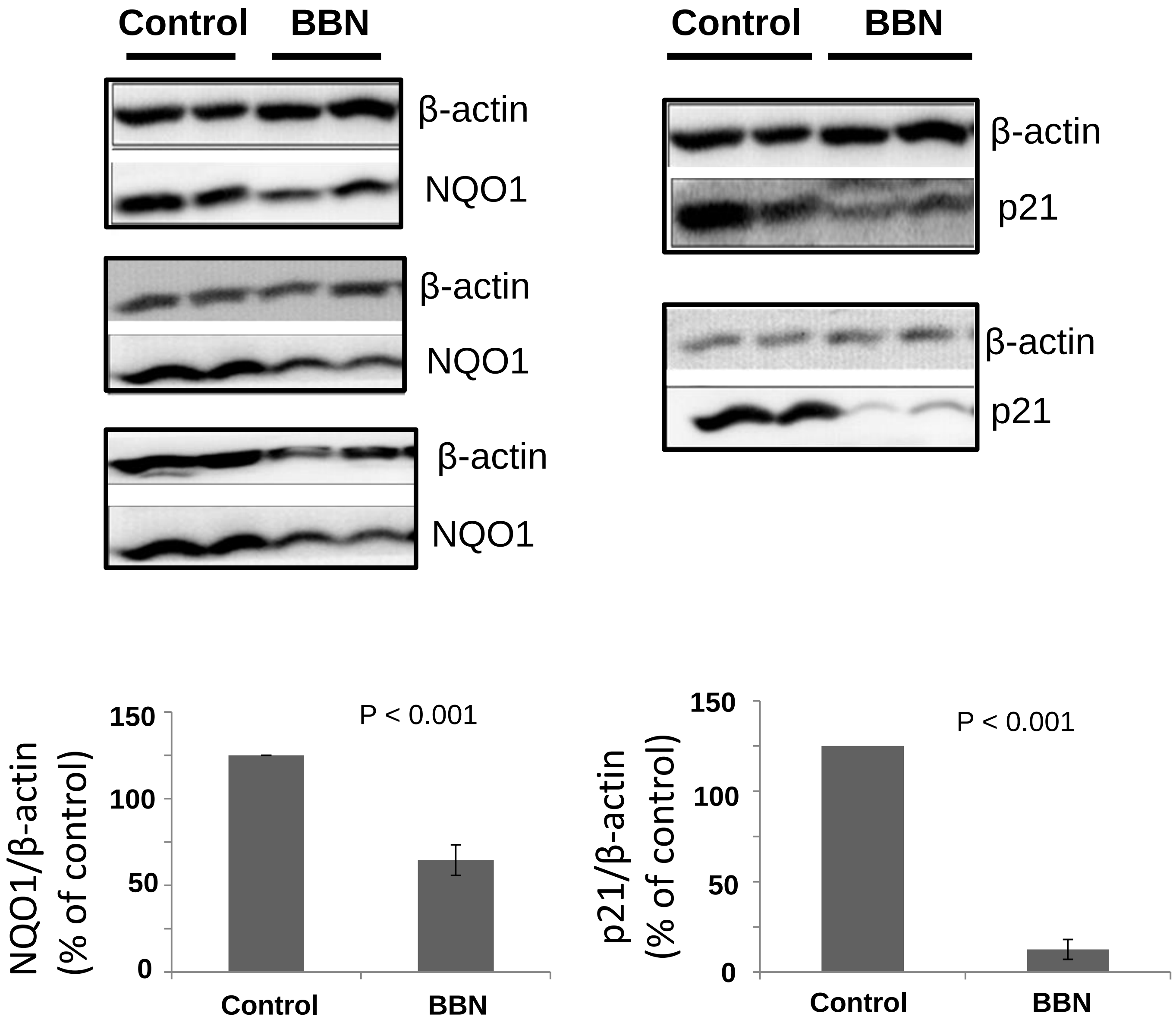

S2 Fig. NQO1 and p21 protein expression of mice bladders with or without 300 ppm BBN treatment for 20 weeks. There are 12 samples (6/group) for NQO1 analysis, and 8 samples (4/group) for p21 analysis. The values shown are mean ± SEM.
