# Supplementary material for: Gene Expression and DNA Methylation Status of Glutathione S-Transferase Mu1 and Mu5 in Urothelial Carcinoma
Source: PLoS One. 2016 Jul 12;11(7):e0159102. doi: 10.1371/journal.pone.0159102 (PMC4942074; doi:10.1371/journal.pone.0159102)
Supplement: S3 Fig — Cells were treated with or without 1.5 mM BBN for 2, 7 and 14 days. (PDF) [file pone.0159102.s003.pdf]

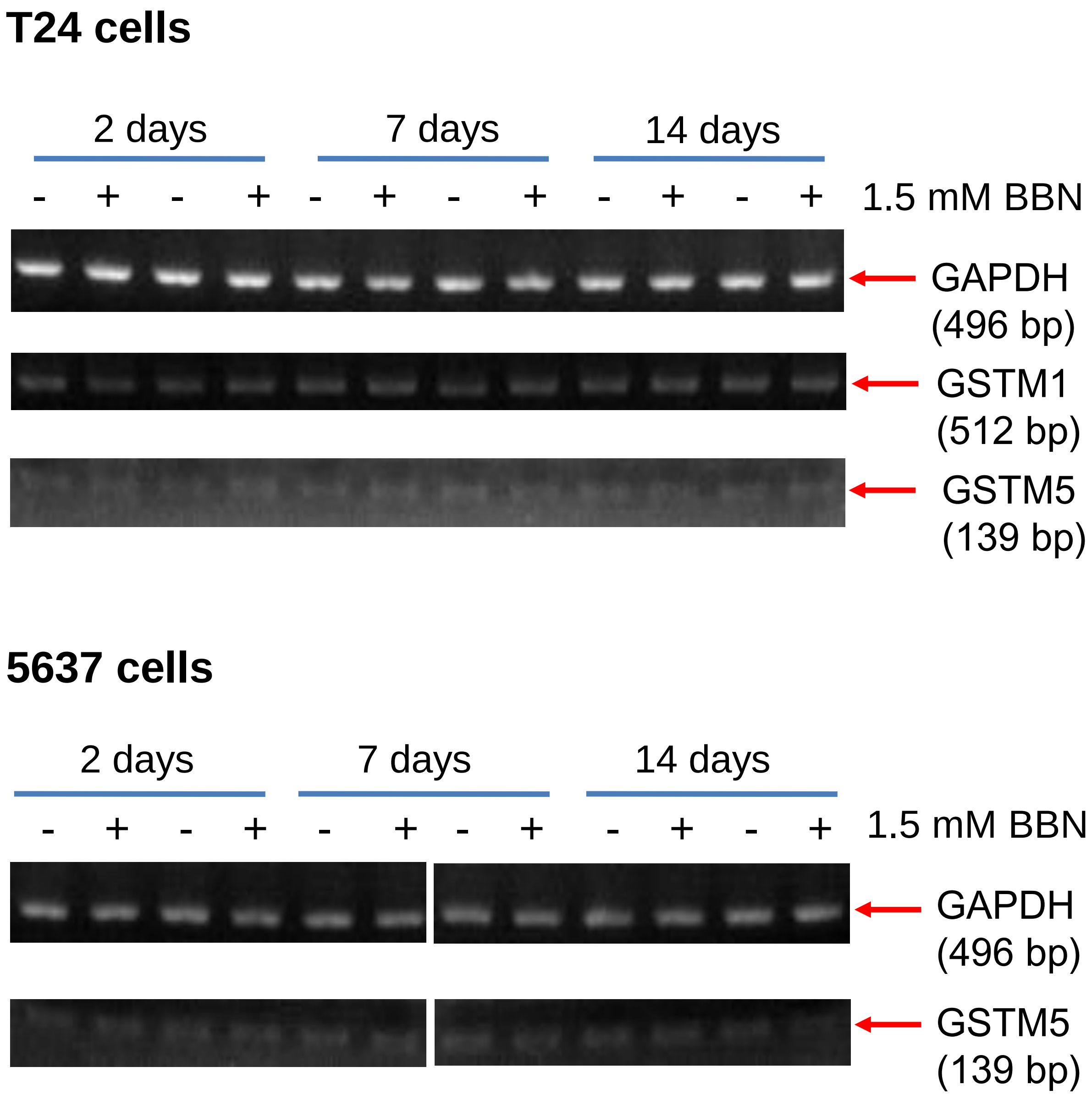

S3 Fig. GSTM1 and GSTM5 mRNA expression of T24 and 5637 cells with or without BBN treatment. Cells were treated with or without 1.5 mM BBN for 2, 7 and 14 days.
